# Supplementary figures and images for: Revisiting the Landscape Mosaic model
Source: PLoS One. 2024 May 29;19(5):e0304215. doi: 10.1371/journal.pone.0304215 (PMC11135788; doi:10.1371/journal.pone.0304215)

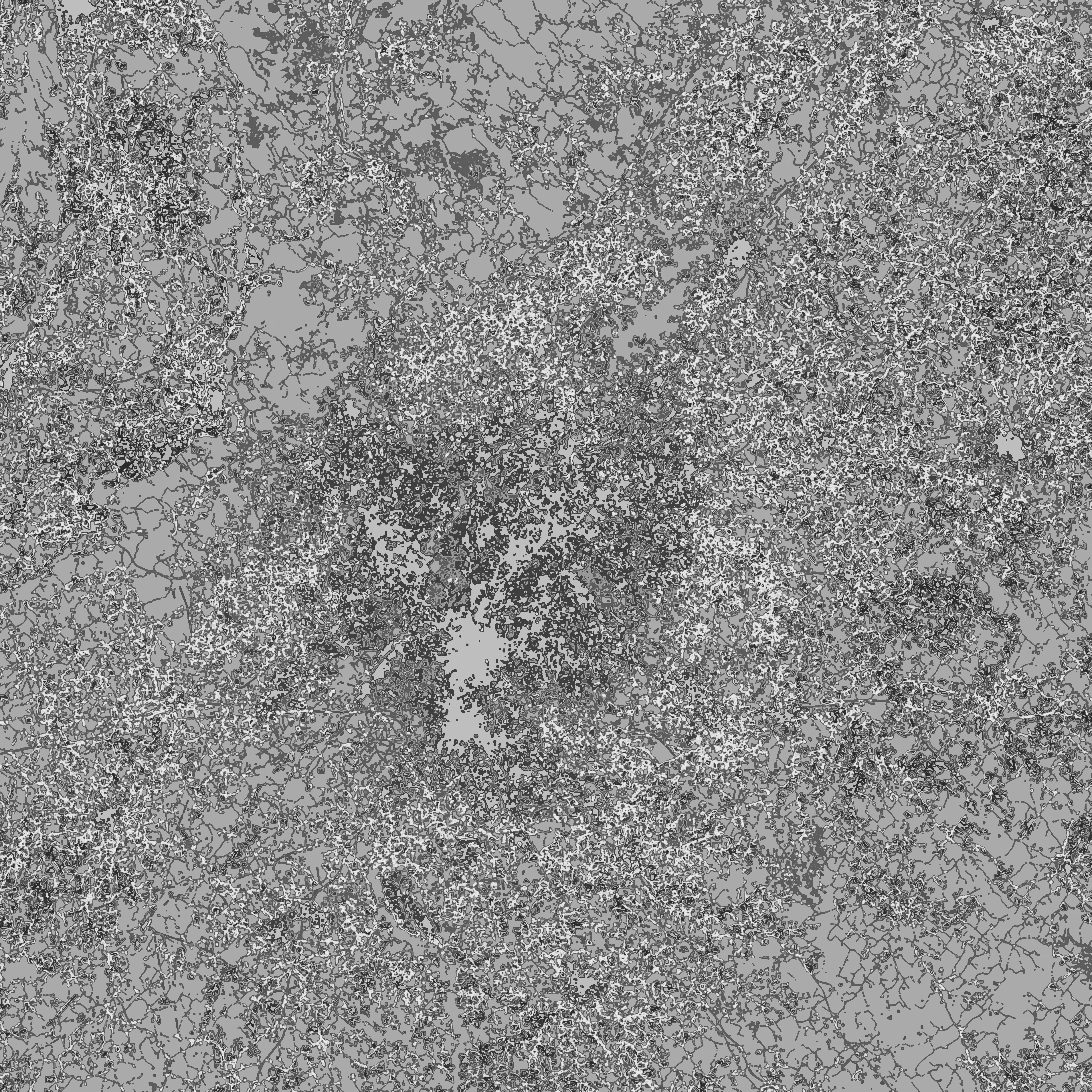

Supplement: S2 File — (ZIP) [file pone.0304215.s002.zip › ATL2001_lm_13/ATL2001_lm_13_103class.tif]

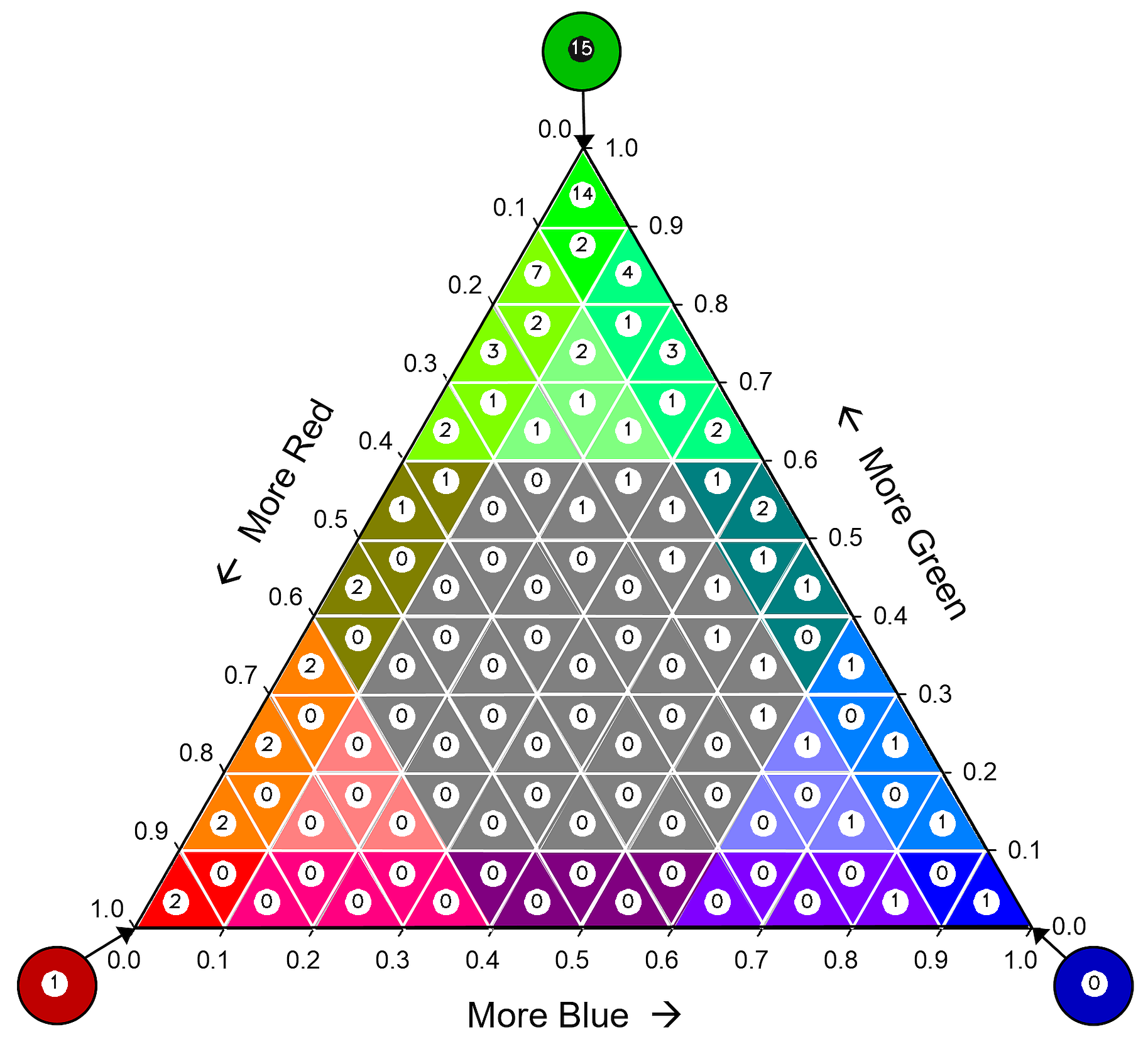

Supplement: S2 File — (ZIP) [file pone.0304215.s002.zip › ATL2001_lm_13/ATL2001_lm_13_heatmap.png]

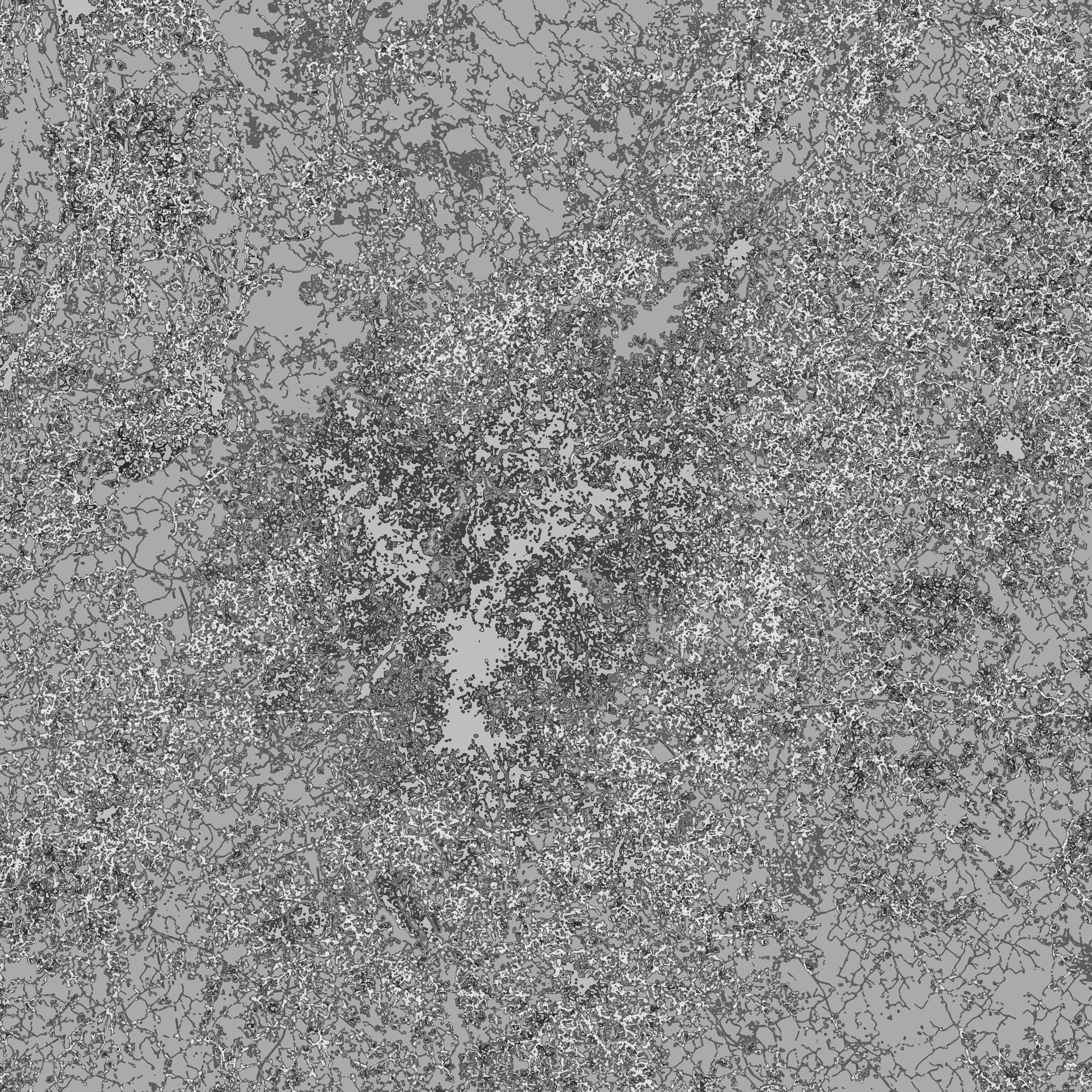

Supplement: S3 File — (ZIP) [file pone.0304215.s003.zip › ATL2021_lm_13/ATL2021_lm_13_103class.tif]

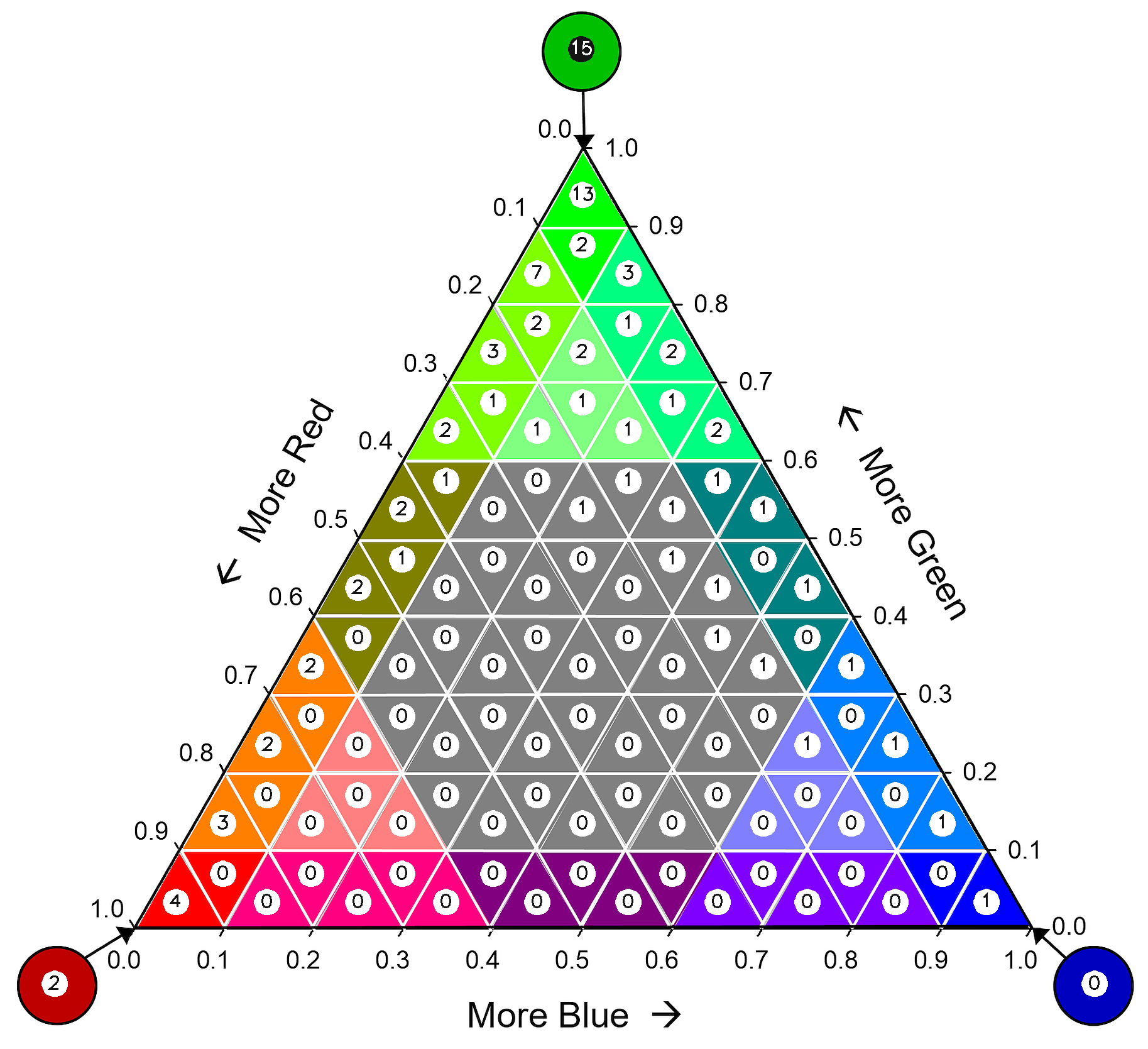

Supplement: S3 File — (ZIP) [file pone.0304215.s003.zip › ATL2021_lm_13/ATL2021_lm_13_heatmap.png]
