# Supplementary material for: Revisiting the Landscape Mosaic model
Source: PLoS One. 2024 May 29;19(5):e0304215. doi: 10.1371/journal.pone.0304215 (PMC11135788; doi:10.1371/journal.pone.0304215)
Supplement: Attachment — Submitted filename: Response to Reviewers.docx [file pone.0304215.s004.docx]

PLoS ONE One Response to Reviewers [PONE-D-23-39102] [EMD:B4940f3297c2ddbc]

**Handling Editor**

The two reviewers provided useful guidance related to this study that the authors should carefully consider during revision. Careful attention in revision with thoughtful reply will be needed to assuage reviewer 2's concern regarding statistical rigor of this study, which if un-addressed, could potentially lead to a rejection. I encourage the authors to submit major revisions to the article and look forward to seeing the revised version.

**Reviewer Response to Journal Questions**

1. Is the manuscript technically sound, and do the data support the conclusions?

The manuscript must describe a technically sound piece of scientific research with data that supports the conclusions. Experiments must have been conducted rigorously, with appropriate controls, replication, and sample sizes. The conclusions must be drawn appropriately based on the data presented.

Reviewer #1: Yes

Reviewer #2: No

2. Has the statistical analysis been performed appropriately and rigorously?

Reviewer #1: Yes

Reviewer #2: No

3. Have the authors made all data underlying the findings in their manuscript fully available?

The [PLOS Data policy](https://gcc02.safelinks.protection.outlook.com/?url=https%3A%2F%2Furldefense.com%2Fv3%2F__http%3A%2F%2Fwww.plosone.org%2Fstatic%2Fpolicies.action*sharing__%3BIw!!DOxrgLBm!FwizbAjHVkuBcg2UrQ9bjsUXCn9zKm9NIOliN9qve57_pJt0bIm7V_hVLy6-E7SExPY8Zwo1OkXtM-HuxM3uNA%24&data=05%7C02%7CWickham.James%40epa.gov%7C00f24e95d79941195b5608dc39066cc7%7C88b378b367484867acf976aacbeca6a7%7C0%7C0%7C638447948492642609%7CUnknown%7CTWFpbGZsb3d8eyJWIjoiMC4wLjAwMDAiLCJQIjoiV2luMzIiLCJBTiI6Ik1haWwiLCJXVCI6Mn0%3D%7C0%7C%7C%7C&sdata=SsQ6hsxxDqskGLfksK2IkUQYiIXscK8PLR7xTNONmuM%3D&reserved=0) requires authors to make all data underlying the findings described in their manuscript fully available without restriction, with rare exception (please refer to the Data Availability Statement in the manuscript PDF file). The data should be provided as part of the manuscript or its supporting information, or deposited to a public repository. For example, in addition to summary statistics, the data points behind means, medians and variance measures should be available. If there are restrictions on publicly sharing data—e.g. participant privacy or use of data from a third party—those must be specified.

Reviewer #1: No

Reviewer #2: Yes

4. Is the manuscript presented in an intelligible fashion and written in standard English?

PLOS ONE does not copyedit accepted manuscripts, so the language in submitted articles must be clear, correct, and unambiguous. Any typographical or grammatical errors should be corrected at revision, so please note any specific errors here.

Reviewer #1: Yes

Reviewer #2: Yes

**Review Comments to the Author**

**Reviewer #1: **General remarks**:**

*The manuscript introduces an improvement to the existing Landscape Mosaic Model and presents an interesting and promising approach to quantify landscape characteristics. However, the manuscript could be improved at several points.*

**Response:** We appreciate the reviewer's clarity and specificity on actions to improve the manuscript.

*First, it is currently unclear to the reader which parts relate to the already existing approach and which parts are newly introduced in the context of this research. This mainly refers to the Methods section.*

**Response:** Excellent point. The Methods section now opens with a clear distinction of past LM model capabilities and the new capabilities introduced in this paper. This is now also described in the Discussion paragraph.

*Second, the manuscript would benefit from a brief but clear introduction of the already existing approach and a following clear description of the improvements suggested here. Generally, the exact steps of the improvements suggested in this manuscript are not fully clear and the Methods section should be improved in terms of clarity.*

**Response:** Thank you, we have rewritten the sections Introduction and Methods accordingly to better distinguish between the existing scheme and the new features that were introduced in our manuscript.

*Third, during the Introduction section, but also during the Discussion section it would be interesting to compare the here introduced methods to already existing approaches to quantify landscape characteristics. Also, the introduction is missing information on why the existing approach needs to be improved.*

**Response:** Thank you for the comment. We have revised the introduction on the rationale for adding new capability to the LM model. We agree that a comparison to other landscape characteristic methods would be interesting, but this should be better addressed in a different paper, while our paper is focused on enhancements to the existing LM model.

*Fourth, the manuscript could be improved by revisiting the Methods and Results section - ensuring a clear separation between the two. Currently especially in the Results section there are several paragraphs which would be helpful in the Methods.*

**Response:** Thank you for the comment. We have revised the methods section to bring more clarity.

*Fifth and last, the manuscript includes quite a few figures and might be easier to approach by readers if some figures are moved to the Supplementary materials.*

**Response:** We now constrain the analysis on Atlanta only. In addition, two figures on scale were merged into one figure. We believe that the current number of figures are needed to explain the features of the enhanced LM product, and to illustrate the new features on reporting, change, and scale.

***Specific remarks**:*

*Line 37: Throughout the manuscript, but especially in the Introduction section, land-cover and land-use are used exchangeable, however, the terms describe slightly different but related aspects of a landscape.*

**Response:** Thank you for spotting this inconsistency. We have revised the text accordingly using the term land cover only and providing a footnote to a book section explaining differences between land cover and land use.

*Line 44: I agree that composition is a crucial point, however, what about configuration?*
**Response:** It is well-established (e.g., Gustafson 1998) that composition and configuration are the fundamental aspects of landscape pattern. It is also well-established that some aspects of configuration can be inferred by measuring only composition (e.g., review by Riitters 2019). In the present case, we can infer juxtaposition (one aspect of configuration) to some extent by measuring the composition in a window. It is possible to perform a fuller analysis of configuration, for example by examining the spatial adjacencies of agriculture, developed, and natural pixels within a window, but then the LM model would not apply.

*Line 47: It might be helpful, especially for readers not familiar with the topic, to briefly explain how remote sensed imagery gets translated in the pixel/cell based LULC maps.*
**Response:** We agree that it is important to inform readership as fully as possible. However, land cover classification is so commonplace now that the reader might find such a discussion of the topic superfluous, even distracting. To reconcile, we included the URL <https://www.sciencedirect.com/topics/earth-and-planetary-sciences/land-use-classification>. Open access links to a wide array of topics by Science Direct seems a good solution to us. Additional information was added in the Data section.

*Line 58 – 65: This section of the manuscript could be shortened and instead readers might benefit more from an improved explanation of landscape mosaic model.*
**Response:** We have revised the sentence beginning on Line 56 (original submission) to “The tri-polar (ternary) classification scheme uses a moving window analysis to assign a LM label to the center pixel of the window based on the proportions of agriculture, natural land, and developed land within the window.” We have added an improved explanation of the landscape mosaic model in the new Methods section.

*Line 63: There is also plenty of literature proposing other methods to quantify landscape structure and patterns.*
**Response:** We agree. However, the objective of the paper is to document updates to the existing Landscape Mosaic model as well as its implementation into a widely used software package (GuidosToolbox). Despite it, we have included in the introduction a review of methods to quantify different landscape metrics citing key references (line 64-73).

*Line 67: So far, no scale-dependent aspects were introduced at all. However, this would be helpful, especially since different scales are explored later.*
**Response:** Thank you for this suggestion. In the revised Introduction section, we have now added an explanation and reference outlining why scale assessments are important, hence justifying the need to address this topic in our manuscript.

*Line 84: What if the landscape cannot be converted into a 3-classes LULC map? This is a strong, simplifying assumption, given the complexity of existing landscapes. Thus, this requires some additional justification.*
**Response:** A ternary classification requires that the sum of the percentages on the three axes is 100%. One feature of the LM implementation is that any of the original classes which do not fit into the 3 primary classes can be coded as “missing data” and thus are ignored in the computation of the percentages for a given window. In that way, any assumptions can be made by the user, and do not require justification here. For example, some of the earlier applications of the LM model set “water” to “missing data” in order to not dilute the signal of development along coastlines. Our paper illustrates the generic approach; the supporting user guide outlines options for re-coding. We also added a paragraph of limitations of the approach at the end of the Discussion section.

*Line 95: Are the sizes of the moving windows based on typical moving window sizes used in previous studies? What is the basis for this selection?*
**Response:** Window sizes are user-specified. We have revised the text to make this point more clear. Various scales are needed to investigate the LM-results across scales and to justify our choices we added the sentence “Our choice of analysis scales was made to span a wide range of scales representing an approximately geometric progression of analysis scales.”

*Line 99-116 and Line 117-129: This paragraph needs some improvement. Currently, it is hard to understand what part of the existing landscape mosaic model is, and what was improved in this manuscript.*
**Response:** We have revised the new Methods section to improve clarity and clearly define the novelty of the new approach in this paper.

*Line 177 – 182: If the extent of the landscape and by that the overall heterogeneity of the landscape has such a big influence of the results, this should be discussed more critically.*
**Response:** We have deeply revised the Introduction, Materials, and in particular Methods section to better explain the new enhancements, which include an analysis at various scales. The latter is important to evaluate various amounts of landscape extent and its local heterogeneity, which is a prime feature of the LM analysis.

*Line 195 – 200: It is unclear what this means. First, scale itself is not extensive (or not), rather different characteristics of the landscape might be more quantifiable at different scales. The three conclusions are very difficult to understand.*

**Response:** The reviewer is correct that landscape patterns are expressed (and perceived) differently at different scales; landscape patterns are scale-contingent. A single-scale demonstration would simplify the manuscript, but a multi-scale demonstration informs readers who may not be interested in the single scale that we arbitrarily select.

*Line 273: What does highly dominant or predominantly mean. What are the exact thresholds to classify this?*
**Response:** We have revised the text adding threshold values to make this concept more clear.

*Line 278: The anthropic context combines two classes, namely agriculture and urban, correct? These two classes have very different characteristics in terms of their habitats, ecosystem services or general importance and value for ecosystems and e.g., biodiversity. For example, species experience an agricultural landscape probably very different than a developed city, even though both are created by humans.*
**Response:** We revised the text to accommodate this valid observation and explain that we illustrate the idea of a generic concept here. The reviewer is correct that agriculture and urban areas are likely to have very different ecological implications. One feature of the LM model is that both anthropic stresses are quantified and mapped using a consistent method. As we illustrate, the weighting of individual partitions of the LM with respect to anthropic context can be easily amended and redefined by the user in the user-specific context.

*Line 287: Even in natural landscapes the anthropic intensity is probably not 0% - in most regions of the world nature was affected at one point or another by humans. For example, especially in central Europe, but also many other parts of the world, even “natural” forests were shaped by human interventions.*
**Response:** Thank you for this important observation, which is correct. No part of the globe is completely free of anthropic effects. That does not obviate the value of the LM model for analyzing a global map that fully characterized everything. One obvious constraint of the LM model is that it cannot take into account tele-coupling (long-distance, indirect effects, perhaps mediated through commerce), but again that does not obviate the value of the LM model for analyzing “local” spatial effects. Moreover, and due to the lack of any additional related information and in particular from a practical perspective, we are confined to the information provided by the land cover map. The methodology we describe is a post-processing methodology, which applies data analysis of the underlying map data. As with any other image analysis, we believe that the user is aware that our methodology is constrained to the map-inherent information and its quality. However, the user could define ‘natural’ in any other way and then use the same LM concept with this new definition of ‘natural’.

*Line 327 (but also others): In general, all presented relative changes of the landscape seem to be rather small (below 10% in many cases). A better contextualization of these magnitudes would be helpful.*
**Response:** We have added a more detailed description to explain why some changes are relatively small, in particular the changes within each of the 103 LM-classes, which are explained by being confined to a very small subspace of the LM chart (line 320).

*Line 348: Configuration seems to be important as well, which gets not captured by the presented approach as far as I understand it.*
**Response:** Please refer to the response to line 44, above.

*Line 358: There are many other tools that help to inform where protected areas should be located. What are the advantages of the approach presented here?*
**Response:** Thank you for this observation. This is true and it could be the topic of a future paper. In our current paper we describe one example application and comparing it to protected area assessment schemes is beyond the scope of this paper. Our paper’s topic is on enhancements of the existing LM. The designation of protected areas can be seen as a post-hoc application of the results (maps) delivered in this study. Aspects such as natural areas scarcity in peri-urban areas and the services they could provide to citizens in urban areas such as recreation opportunities, clean air, climate regulation, are important topics. However, this is beyond the scope of this paper. Another interesting application of the LM model could be to examine the scale(s) at which a given protected area is actually “protected” For example, a small protected area could be free of anthropic stress in a small window, yet contained within a larger landscape (window) that was completely urbanized. In an earlier paper (Riitters et al 2018, <https://doi.org/10.3390/f9110723> ) we addressed a similar question of the efficacy of “protection” with regard to invasive plants, using a simplified version of the LM model. A decision about where to locate a new protected area could consider this aspect, which is also related to the size of the proposed protected area.

*Line 368: Identify the WUI using the approach presented in this manuscript will only be possible if values can be connected to known WUI areas (so basically a ground-truthing or training dataset which puts the WUI in relation to outputs of the approach).*
**Response:** Yes, this again could be material for an additional paper. However, it would be distracting here to go into more detail on WUI since the focus of our paper is on the enhanced LM product. There is not a general definition of “WUI” based on land cover alone. In the US, the “official” national definition is articulated at the census “block” level, and combines forest cover data with housing density data, and ignores agriculture. We think the LM could be a starting point for another WUI definition that also incorporates agriculture (WUAI).

*Line 376: This sentence seems out of context.*

**Response:** Thank you, we agree and this sentence has been removed.

**Reviewer #2**

*This article provides an interesting study of landscape context using the landscape mosaicking technique based on the time-series of NLCD land cover classification maps. The study offers an enhanced assessment of land cover class changes and distributions over various sizes of neighborhoods for three types of land use – agriculture, natural, and urban.*

**Response:** We appreciate the reviewer’s thoughtful review of the paper.

*In my opinion, the main weaknesses of this study are the statistical principles used for data aggregation and analysis, and the absence of validation. The proportions of pixels in the neighborhood were estimated using generic pixel counting without taking into account omission and commission errors. This approach undermines the main strength of the study (the use of a 103-class scheme that enhances thematic context) by introducing high level of errors in the proportion-based taxonomy. Moreover, the study does not provide any validation of the produced products or any assessment for the estimated statistics.*

**Response:** We understand the reviewer’s appreciation of the importance of statistically rigorous accuracy assessment of land cover data. One of the reasons NLCD was chosen as the database to demonstrate the new enhanced LM-product was the high quality of the NLCD data, demonstrated by previous statistically rigorous accuracy assessments of these data. NLCD accuracy assessments were built on the good practice recommendations in Stehman and Czaplewski (1998) and further recommendations outlined in Olofsson et al. (2014) for all NLCD accuracy assessments published after Olofsson’s article. For example, all NLCD accuracy assessments published after Olofsson et al. (2014) include a map bias column in the error matrices to quantify pixel counting bias. We published work on sensitivity of land cover metrics to land cover misclassification about 25 years ago (PE&RS, 1997, 64:397-402) Given the objectives of the paper, a sensitivity analysis is outside the scope of this paper. We’ve added a paragraph in the Data section about the relevance of map bias to the application of the enhanced LM-product introduced in this manuscript.

NLCD accuracy assessment:

Stehman, Wickham et al. NLCD 1992 accuracy <https://doi.org/10.1016/S0034-4257(98)00010-8>

Wickham, Stehman et al. NLCD 2001 accuracy <https://doi.org/10.1016/j.rse.2010.01.018>

Wickham, Stehman et al. NLCD 2006 accuracy <https://doi.org/10.1016/j.rse.2012.12.001>

Wickham, Stehman et al. NLCD 2011 accuracy <https://doi.org/10.1016/j.rse.2016.12.026>

Wickham, Stehman et al. NLCD 2016 accuracy <https://doi.org/10.1016/j.rse.2021.112357>

Wickham, Stehman et al. NLCD 2019 accuracy <https://doi.org/10.1080/15481603.2023.2181143>

Research supporting NLCD accuracy assessments:

Stehman, Czaplewski 1998 *Remote Sensing of Environment*, 64, 331−344

Stehman, Wickham 2011 <https://doi.org/10.1016/j.rse.2011.06.007>

Stehman, Foody 2019 <https://doi.org/10.1016/j.rse.2019.05.018>

Stehman, Wickham 2020 <https://doi.org/10.1007/s10980-020-01029-1>

*More detailed comments:*

*1. In the abstract, please add the institution that provides NLCD to be more specific.*
**Response:** Revised as recommended.

*2. L52: Please specify the projection of the maps*
**Response:** Revised as recommended.

*3. I think the introduction should be extended. Authors should provide more details on the impact of land cover/land use on the environment, the segmentation of land cover, integration into monitoring systems at the country or regional level, and policy-making. Most importantly, they should focus on the Landscape Mosaic concept and provide specific examples and benefits of its use. The introduction lacks a literature review.*
**Response:** We appreciate the reviewer’s concern. However, we are not sure how we can improve upon the literature review provided in the original submission. In the original submission, we succinctly review use of the LM model, identify eight applications, and cite 12 sources. Four of the citations reference EU or USA applications endorsed by government agencies or US national NGO, which are relevant to the discussion of applications in real-world problems.

*4. The images provided by the authors in the article have a resolution that is too low.*
**Response:** In the revision, we have reduced the total number of figures and enhanced the resolution. High-resolution versions of all figures will be provided for the production phase. Full resolution maps. heat maps, and statistics are provided in the supplemental files S1-S3.

*5. I recommend separating the Materials and Methods section into two separate sections. One section should describe the used dataset, while the other should outline the processing and analysis methods.*
**Response:** Agreed. The manuscript is revised accordingly.

*6. L196-200, please provide quantification for the stated facts.*
**Response:** Thank you. Revised as recommended.

*7. In the section “change in land cover context” please provide all numbers in the same numerical formats and with the same precision*
**Response:** Thank you. Revised as recommended.

*8. It is bad practice to estimate proportions or areas for land cover classes, and especially land cover changes, using generic pixel counting. The maps used have omission and commission errors, and aggregating the time-series of the land cover maps into classes based on the percentage of pixels in the neighborhood can lead to a high level of errors and incorrect statistical analysis. The 30 m remote sensing-based land cover classification maps have numerous problems (mixed pixels, image mis-registration, classification model errors) that do not allow for unbiased estimation of areas/proportions [1]. Thus, authors should either integrate statistical information from the maps' validation reports into their analysis or use a sample-based stratified approach for area estimation [2,3].*

*References:*

*1. Olofsson, P. et al. Mitigating the effects of omission errors on area and area change estimates. Remote Sens. Environ. 236, 111492 (2020).
2. Olofsson, P. et al. Good practices for estimating area and assessing accuracy of land change. Remote Sens. Environ. 148, 42–57 (2014).
3. Cochran, William Gemmell. Sampling techniques. john wiley & sons, 1977.*

**Response:** We share the reviewer’s concern about map bias. NLCD 2019 map bias estimates for forest, shrubland, grassland, and wetland, the main components of the natural class in the LM model, were -2.34% , 1.38%, 0.09%, and 1.37%, respectively (p. 10 [doi: 10.1080/15481603.2023.2181143](https://doi.org/10.1080/15481603.2023.2181143)) (NLCD 2021 results not finished). Of course, these estimates are based on a sample and therefore difficult to extrapolate to a census (map). To push back somewhat, as the reviewer well knows, an assumption underpinning map bias is error-free reference data. A necessary but unrealistic assumption that propagates to estimation of map bias. As an aside, incorporating reference error into the assessments of NLCD 2021 thematic accuracy was investigated,(e.g., <https://doi.org/10.1016/j.rse.2021.112806>). We added a paragraph on map bias and how it relates to LM model results in the revised methods section.

*9. No uncertainty analysis, confidence intervals, p-values, or accuracies were provided in this study.*

**Response:** A map showing the confidence interval for each pixel isn't really possible because the LM is a classification (a categorical variable) while a confidence interval requires numerical data (a continuous variable). Moreover, the topic of our manuscript is to introduce additions to an already existing generic, shifting neighborhood analysis methodology. As any other moving window analysis, such an approach is based on pixel counting, which by definition depends on the quality of the input data used. We used NLCD as an example but could have used any other land cover dataset as well. The scope of our paper is to illustrate the post-processing methodology and potential benefits for application. While valid, it is beyond the scope of our paper to assess the accuracy of the LM-results, which are proportional to the quality of the input dataset. We believe this is clear to the reader and evaluating it would be distracting from the actual scope of our paper.

*10. L351-355 please provide more details about specific of the generated information use*

**Response:** We believe that the application potential of the introduced enhanced LM-product as outlined in the revised Conclusions section addresses this question.

*11. The authors should also provide more information on the aggregation of NLCD classes into the three classes used. Especially important details should be provided regarding the differences between land cover and land use. For instance, an agriculture field can remain unsaved for several years with extensive cover of natural vegetation before the season when it will be planted. As a result, land cover maps can show the change from cropland to natural vegetation; however, in terms of land use and your taxonomy, it is still considered agriculture.*

**Response:** The aggregation into three classes follows the examples provided by earlier applications of NLCD data in landscape pattern analysis. NLCD is a mixed land use/cover classification and is a property of the data which must be understood by users. Of course, all types of land cover and land use can change over time, and our objective was to demonstrate how that results in a shifting landscape mosaic over time. In any case, a data user may consider somehow adjusting the input data to account for the reviewer’s concerns but that would obviate the replicability of the paper’s findings.

*12. Authors should provide discussion of the weaknesses of the proposed approach and problems of the generated data.*

**Response:** Thank you for this suggestion. We have now included a paragraph on the limitations of the approach at the end of the Discussion section.
