# Supplementary material for: Revisiting the Landscape Mosaic model
Source: PLoS One. 2024 May 29;19(5):e0304215. doi: 10.1371/journal.pone.0304215 (PMC11135788; doi:10.1371/journal.pone.0304215)
Supplement: Attachment — Submitted filename: Response to Reviewers.docx [file pone.0304215.s005.docx]

PLoS ONE One Response to Reviewers [PONE-D-23-39102R1]

**Reviewer #1: General remarks:**

*The manuscript improved a lot from the previous version. Especially the Introduction and the Methods now clearly introduce the general LM concept and how it is improved in this manuscript. It is now much easier for readers to follow the here presented research. Additionally, the manuscript benefits from moving some of the figures to the Supplementary Materials and now more focus is put on the main figures. Last, the newly added limitations will help readers to assess if the unquestionable valuable approach is fitting for their research question.*

*While the improvements of the existing LM approach to 103 class output is undoubtfully a very valuable and interesting contribution to the field, there also might be situations and advantages of the existing 19 class output approach. One that comes to mind is an easier interpretation and communication of the results, especially for a non-scientific community. While I acknowledge that this is outside the scope of this manuscript, a very brief discussion of this could be valuable.*

**Response:** We appreciate the reviewer's clarity and specificity on actions to improve the manuscript. We added a short sentence (line 403 in the revised version) to clarify that the enhanced 103-class model also includes the information of the original 19-class model.

***Specific remarks**:*

*Line 74: I would suggest rephrasing this sentence using present tense (as in the following text in the paragraph) to highlight that these improvements are presented in this manuscript.*
**Response:** Changed to present tense as suggested (line 74 in the revised version).

*Line 122: While it is discussed and mentioned later in the manuscript that the classification into the three classes depends on the research questions, data availability, … it might be helpful for readers to already give a brief outlook/reference to this later in-depth discussion already here in the manuscript.*
**Response:** Thank you. We have now added this comment and a reference to the Discussion section (line 123 in the revised version).

*Line 222: Does the moving window have to be a square, or are also approximated circles (within the raster context) possible?*
**Response:** Conceptionally, the moving window can be of any shape. From a practical perspective, as is often the case in spatial analysis using raster maps, the moving window ([kernel](https://en.wikipedia.org/wiki/Kernel_(image_processing))) is of square shape and of size *n* x *n* pixels (in our study, n = 7, 13, 27, 81, or 243).

*Line 266: Similar as with the previous comment, I think the latest version of the manuscript benefits from adding a discussion about the anthropogenetic impact on most natural ecosystems later in the Discussion section. Yet, I would again suggest to already briefly mention this here and reference to the Discussion.*
**Response:** Thank you, we have added a reference to the Discussion section (line 266 in the revised version).

*Line 360: I cannot fully follow the reasoning about “human activity tends to be locale in scale but also spatially pervasive” as these two are rather contradicting. Furthermore, one interesting point might be that the first natural habitat could also be understood as an absence of human impacts, whereases the other two classes are a direct result of human impacts (urban and agriculture).*
**Response:** The reasoning is that human activity starts at local scale, but its influence also impacts other non-local scales. We rephrased the line accordingly in the revised version and added the observation you suggested in line 367 ff of the revised manuscript, thank you for this suggestion.

**Reviewer #2**

*The authors conducted great work in responding to my comments, and I am satisfied with most of the edits and responses. The only thing with which I do not agree is the assumption about 'error-free reference data.' Proportion estimations and area estimations are important parts of creating and using land cover products. However, at the same time, I fully understand the authors' point regarding why they do not want to extend their experiment with additional sampling, validation, and re-calculations. It is outside the scope of their particular improvement. However, it is still very important for the authors to include in the discussion a section that describes best practices for validation, area, and proportion estimations in the context of Landscape Mosaic Models use.*

**Response:** Thank you for this suggestion. It would have been interesting to explore this aspect more in depth. However, this would not be possible because this topic is beyond the scope of our paper. Nevertheless, as suggested, we included a new paragraph in the Discussion section addressing the suggested points (line 393 – 396 in the revised version).
